# Gastrointestinal Bleeding and Diffuse Skin Thickening as Kaposi Sarcoma Clinical Presentation

**DOI:** 10.1155/2015/424508

**Published:** 2015-12-10

**Authors:** Sara Querido, Henrique Silva Sousa, Tiago Assis Pereira, Rita Birne, Patrícia Matias, Cristina Jorge, André Weigert, Teresa Adragão, Margarida Bruges, Domingos Machado

**Affiliations:** ^1^Department of Nephrology, Centro Hospitalar do Médio Tejo, Avenida Xanana Gusmão, Apartado 45, 2350-754 Torres Novas, Portugal; ^2^Department of Nephrology, Centro Hospitalar de Lisboa Ocidental, Avenida Professor Reinaldo dos Santos, 2790-134 Carnaxide, Portugal

## Abstract

A 56-year-old African patient received a kidney from a deceased donor with 4 HLA mismatches in April 2013. He received immunosuppression with basiliximab, tacrolimus, mycophenolate mofetil, and prednisone. Immediate diuresis and a good allograft function were soon observed. Six months later, the serum creatinine level increased to 2.6 mg/dL. A renal allograft biopsy revealed interstitial fibrosis and tubular atrophy grade II. Toxicity of calcineurin inhibitor was assumed and, after a switch for everolimus, renal function improved. However, since March 2014, renal function progressively deteriorated. A second allograft biopsy showed no new lesions. Two months later, the patient was admitted due to anuria, haematochezia with anaemia, requiring 5 units of packed red blood cells, and diffuse skin thickening. Colonoscopy showed haemorrhagic patches in the colon and the rectum; histology diagnosis was Kaposi sarcoma (KS). A skin biopsy revealed cutaneous involvement of KS. Rapid clinical deterioration culminated in death in June 2014. This case is unusual as less than 20 cases of KS with gross gastrointestinal bleeding have been reported and only 6 cases had the referred bleeding originating in the lower gastrointestinal tract. So, KS should be considered in differential diagnosis of gastrointestinal bleeding in some kidney transplant patients.

## 1. Introduction

Kaposi's sarcoma (KS) was first described in 1872 as an unusual haemorrhagic cutaneous lesion [1]. It is known as a rare tumour comprising 0.1% of all malignancies worldwide, with an increased incidence in transplant recipients [2, 3]. In these patients, it has an incidence about 400–500 times higher than in general population [4], comprising 0.5–0.7% of malignancies that occur in organ transplant recipients [5–8]. Infection with Kaposi's sarcoma-associated herpesvirus (KSHV, commonly known as human herpesvirus type 8, HHV-8) is required for the development of this sarcoma [9]. The wide variation in incidence has been attributed to populations' characteristics [9, 10] and to immunosuppression regimen in organ recipients [11].

Skin lesions are the most common manifestation in patients with KS, although mucosal sites, lymph nodes, and viscera can also be involved [12]. Visceral involvement occurs in less than 50% of patients [13, 14] and is considered a systemic multifocal progressive tumour of the reticuloendothelial system [15]. The most frequent location for KS visceral involvement is the gastrointestinal tract. The small intestine is the most frequently affected area, followed by the stomach, oesophagus, and, lastly, colon [13]. However, the disease is usually asymptomatic as the tumour grows primarily in the submucosa [16]. Therefore, the disease commonly produces no symptoms, namely, anaemia, vomiting, diarrhoea, or intestinal obstruction or perforation [16]. Gastrointestinal bleeding requiring blood transfusions is also rare [16–18].

We report a case of KS in a renal transplant recipient with low cumulative exposure to immunosuppression, presented as lower gastrointestinal bleeding with rapid progression to death thirteen months after receiving a kidney allograft.

## 2. Case Presentation

A 56-year-old African man, from Guinea-Bissau, received a kidney from a deceased donor with 4 HLA mismatches in April 2013. The aetiology of his chronic kidney disease was unknown and he had been on haemodialysis for five years. In 2012, he suffered acute upper gastrointestinal bleeding; an endoscopy showed no lesions.

The recipient presented 0% panel reactive antibodies (PRA) and no anti-HLA class I and II antibodies; donor and recipient were both cytomegalovirus (CMV) IgG positive.

He received initially basiliximab and the maintenance immunosuppressive regimen was achieved with tacrolimus, mycophenolate mofetil (MMF), and prednisone.

Immediate diuresis and progressive improvement of renal function (creatinine 1.34 mg/dL at discharge) were observed in the postoperative period.

In October 2013, unexpectedly, the serum creatinine level increased to 2.57 mg/dL. Doppler ultrasonography showed no alterations. A renal allograft biopsy revealed interstitial fibrosis and tubular atrophy grade II, assumed as toxicity of calcineurin inhibitor. His medication was switched to everolimus and serum creatinine levels slowly decreased until serum creatinine of 1.8 mg/dL.

In March 2014, the patient was admitted due to anasarca (serum creatinine of 3.28 mg/dL and proteinuria of 395 mg/day). New renal allograft biopsy was carried out and showed no additional changes. Anti-HLA class I and II antibodies remained negative. mTOR inhibitor was stopped, and the patient was, once again, treated with calcineurin inhibitors with no improvement of renal function.

In May 2014, the patient was admitted due to anuria with significant deterioration of renal function (serum creatinine of 6.9 mg/dL), haematochezia, and anaemia (haemoglobin: 7.5 g/dL), requiring 5 units of packed red blood cells. Extremities swelling due to bilateral oedema and diffuse, ill-defined thickening of the skin and deeper tissue of the limbs were also present. No mucosal lesions were identified.

**Figure 1:**
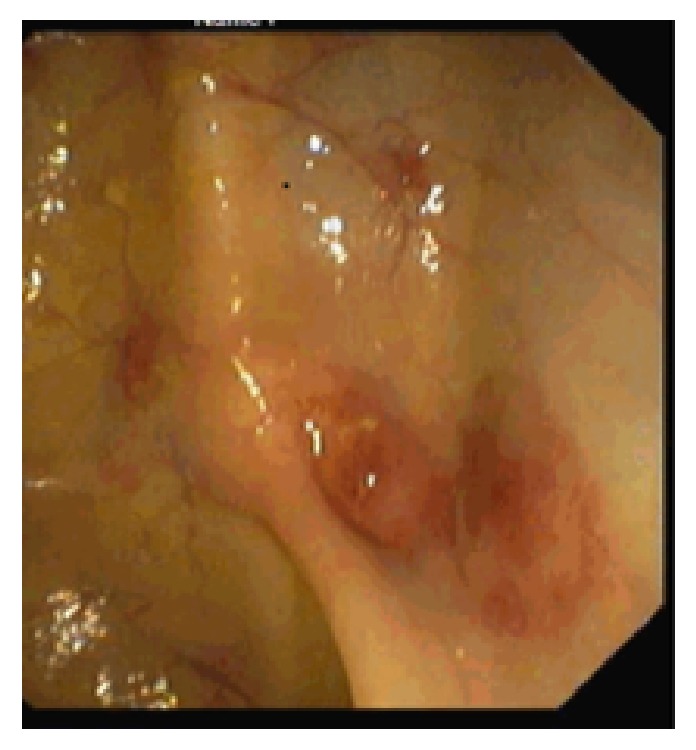
Colonic mucosa showing haemorrhagic patches.

Colonoscopy showed haemorrhagic patches in the colon and the rectum (Figure 1). Histology confirmed proliferation of spindle cells with vascular spaces slit and positivity for CD-31 and HHV-8, confirming gastrointestinal KS. A skin biopsy revealed cutaneous involvement of KS. The investigation of graft failure was inconclusive, immunosuppressive therapy was progressively stopped, and haemodialysis was started.

In few days, the patient had substantial clinical deterioration with multisystem organ failure, leading to death in June 2014. No necropsy was allowed.

## 3. Discussion

KS is a multicentric and angioproliferative tumour with an increased incidence both in organ transplant recipients, due to immunosuppression, and in AIDS patients. Independent determinants of KS development are age, gender, and immunosuppressive protocol, including induction therapy [19]. The literature reveals that KS is found 6.5–20 months after renal transplantation [20, 21], with higher prevalence in patients who have undergone cyclosporine-based immunosuppressive protocols [22]. Our patient, who had never been under cyclosporine, developed KS 13 months after undergoing transplantation, besides the low cumulative immunosuppressant exposure.

Previous reports showed that the disease has gastrointestinal involvement in 40 to 48% of patients [13, 14], commonly due to lesions in the upper gastrointestinal tract, whereas large bowel is rarely affected [23]. Initially, gastrointestinal KS manifests itself with few or no symptoms. However, rarely, it may present with anorexia, weight loss [1, 14, 24], gastrointestinal bleeding, diarrhoea, or intestinal obstruction or perforation [14].

Our case, with severe colonic involvement and bleeding, requiring multiple blood transfusions, is unusual as only 18 cases of KS with gross gastrointestinal bleeding have been reported [25–27] and in only 6 cases [24, 25] that bleeding was due to involvement of the lower gastrointestinal tract. Among these, just 4 cases of gastrointestinal bleeding occurred in renal transplant recipients. Only one case of lower intestinal bleeding due to KS in kidney transplant recipients was previously reported [24].

Endoscopically, different KS lesions have been described: haemorrhagic patches, discrete papules, volcano-like lesions with central umbilication, and large exophytic lesions projecting into the lumen [25]. Histology usually reveals proliferating spindle cells, poorly defined vascular channels with positivity for HHV-8, CD-31, and/or CD-34 [25], known as lymphatic endothelial cell markers [28]. This pattern matches the observations in our case.

No specific treatment for KS is nowadays available. Despite the risk of graft rejection, immunosuppressive drugs reduction has been recommended. Discontinuation of calcineurin inhibitors and switch to mTOR inhibitors due to their antiproliferative properties are possible strategies [29]. Chemotherapy with vincristine, paclitaxel, or liposomal anthracyclines and radiotherapy are other possible therapies [30]. In this patient, the clinical course was fulminant and these measures were clinically unsuitable.

Data concerning survival are not consistent, although prognosis seems to be worst in transplant recipients with visceral involvement. Nevertheless, the rapid progression to death is not a common denouement of KS.

Besides that, KS with gastrointestinal involvement should be considered in the differential diagnosis of gastrointestinal bleeding in some renal transplant patients.
